# The Interaction Between Typically Developing Students and Peers With Autism Spectrum Disorder in Regular Schools in Ghana: An Exploration Using the Theory of Planned Behaviour

**DOI:** 10.3389/fpsyg.2021.752569

**Published:** 2021-09-22

**Authors:** Maxwell Peprah Opoku, William Nketsia, J-F, Wisdom Kwadwo Mprah, Elvis Agyei-Okyere, Mohammed Safi

**Affiliations:** ^1^Department of Special Education, College of Education, United Arab Emirates University, Al-Ain, United Arab Emirates; ^2^School of Education, Western Sydney University, Penrith, NSW, Australia; ^3^Faculty of Education, Crandall University, Moncton, NB, Canada; ^4^School of Public Health, Kwame Nkrumah University of Science and Technology, Kumasi, Ghana; ^5^Department of Planning, Kwame Nkrumah University of Science and Technology, Kumasi, Ghana

**Keywords:** Autism Spectrum Disorder, inclusive education, typically developing students, theory of planned behaviour, Ghana

## Abstract

The purpose of this study is to assess the intention of typically developing peers towards learning in the classroom with students with Autism Spectrum Disorder (ASD). In developing countries, such as Ghana, the body of literature on the relationship between students with disabilities and typically developing peers has been sparsely studied. Using Ajzen's theory of planned behaviour as a theoretical framework for this study, 516 typically developing students completed four scales representing belief constructs, attitudes, subjective norms, and perceived behavioural controls (self-efficacy), hypothesised to predict behavioural intention. The data were subjected to a *t-test*, analysis of variance, and structural equation modelling. The modelling confirmed the combining ability of attitude, subjective norms, and perceived behavioural controls to predict intention. We conclude by revealing the need for policymakers to consider designing programmes aimed towards promoting social relationships between students with ASD and typically developing peers.

## Introduction

Autism Spectrum Disorder (ASD) is one of the most common types of developmental disorders globally (Malcolm-Smith et al., 2013). Autism is a developmental disability with its main feature being the presence of persistent impairments, which leads to poor social relations, repetitive behaviours or interests in a particular activity (Baio et al., 2018; Maenner et al., 2020). Globally, it is estimated that at least 1% of the population is living with ASD (Malcolm-Smith et al., 2013). The cause of ASD is unknown, and there are various symptoms and levels of functioning among individuals living with a disability (American Psychiatric Association, 2013; Maenner et al., 2020). One of the foundational symptoms of ASD is social-communication difficulties, such as the inability of diagnosed persons to follow or provide appropriate responses in conversations, to read verbal, and non-verbal cues and to develop friendships with peers (American Psychiatric Association, 2013; Hossain et al., 2017; Onaolapo and Onaolapo, 2017). While there is a rise in ASD prevalence (Onaolapo and Onaolapo, 2017), empirical studies from developing countries in sub-Saharan Africa, such as Ghana, remain small, and sometimes non-existent (Malcolm-Smith et al., 2013; World Health Organisation, 2013; Hossain et al., 2017; Onaolapo and Onaolapo, 2017), requiring significant further scholarly insight and study.

The challenges that people living with ASD face in society cannot be overemphasised (e.g., Han and Cumming, 2019; Martin et al., 2019). Including students with ASD in regular classrooms is considered an effective approach to promoting their societal acceptance (Keane et al., 2012; Ashman, 2015). For instance, previous studies have reported that the participation of students with ASD in regular classrooms enhances their social acceptance and academic performance (Chan and O'Reilly, 2008; Finke et al., 2009; Banda et al., 2010; Eldar et al., 2010; Locke et al., 2010; Keane et al., 2012; Martin et al., 2019). Specifically, typically developing students (students without ASD) become role models for students with ASD, who learn especially socially desirable behaviours from general students (Ashman, 2015; J-F et al., 2020). Alternatively, typically developing students learn to understand peers with ASD and support them in acquiring academic and social skills (Banda et al., 2010; Lindsay et al., 2014). This probably lends support to understanding the intention of typically developing students towards peers with ASD in Ghana, which is in the early stages of practising inclusive education.

Ghana is a West African country located along the Atlantic Ocean and the Gulf of Guinea with an estimated population of 30 million. The government recognises education as essential to promoting economic growth and national development. The three-tier education structure comprised 11 years of basic education (2 years of kindergarten, 6 years of primary education and 3 years of junior secondary education), 3 years of senior secondary education, and 2–6 years of tertiary education. Basic and senior secondary education are free and compulsory for all children between 6 and 16 years old (Ministry of Education, 2016). Therefore, reasonable adjustments and accommodations need to be created for students with ASD to ensure their equitable access to and participation in all levels of education.

There is an intersection between disability and cultural beliefs (e.g., Baffoe, 2013; Kassah et al., 2014, 2018; Ametepee and Anastasiou, 2015) in Ghana, which has had negative ramifications for efforts to achieve equitable access to education for all (Anthony, 2011; Kassah et al., 2018). In traditional Ghanaian society, many hold deep-rooted negative beliefs about spiritual influences causing disabilities. ASD is widely understood as “madness,” and children with the disorder are sometimes called “children from rivers”; hence, this group of individuals with disabilities is sometimes labelled “idiots” (Anthony, 2011; Baffoe, 2013). Consequently, children with ASD are often victims of marginalisation and discrimination and are side-lined from all activities in their communities (Anthony, 2011; Ametepee and Anastasiou, 2015). Thus, a national commitment to promoting the learning of all children and the acceptance of children with ASD, especially by their typically developing peers, is vital in enhancing their well-being, and participation in education and society at large. While the global burden of ASD is very high (Hossain et al., 2017; Onaolapo and Onaolapo, 2017), official policy documents in Ghana do not mention the prevalence of such disorders. In fact, the Ghana Statistical Service (2012) erroneously categorised ASD as a form of intellectual disability, which seems to have affected scholarly interest in the welfare of individuals living with ASD in Ghana. This inconsistency and misinformation provide strong justification for studying the interaction between students with ASD and their typically developing peers in secondary schools in Ghana to possibly trigger a national discourse on an appropriate response. Therefore, in this study, we aim to examine the intention of typically developing secondary school students towards learning in the same classroom with peers with ASD in Ghana. The study has potential to provide useful guidelines on best way to promote the acceptance of students with ASD in schools.

### Theoretical Framework

Beliefs have been found to be critical to understanding the interaction between individuals and social phenomena (Büssing et al., 2019; Wang et al., 2021). In particular, individuals live in a nested system where events in society affect their well-being (Büssing et al., 2019; Wang et al., 2021). Ajzen's (1991) theory of planned behaviour (TPB) captures all the important beliefs that explain an individual's intention towards a given behaviour. The theory extends the theory of reasoned action (TRA), which describes behaviour as a product of two related beliefs: behavioural and normative (Fishbein and Ajzen, 1975). While behavioural beliefs refer to an individual's perception of the outcome of a behaviour, normative beliefs refer to the influence of social pressure on an individual regarding performing a given behaviour (Ajzen, 2011). The expectation-value logic explains the relationship between beliefs and intention towards a behaviour. A behavioural belief emanates from the opportunity one has to perform a behaviour. The person evaluates the behaviour, and anticipates the outcome. If a behaviour is perceived favourably, an individual will demonstrate positive intention, and behaviour. The reverse is true when the individual perceives the behaviour unfavourably.

Normative beliefs refer to one's perception of how individuals within their social circle support a given behaviour (Ajzen, 1991). If the social pressure affecting the behaviour is perceived positively, the individual will be positive towards the behaviour. Both behavioural and normative beliefs relate to actions one has control over or the power to decide upon. However, Ajzen noted the important role played by factors that individuals have no control over in the performance of behaviour. Ajzen (2011) expanded the theory of reasoned action (TRA) and argued that there might be a third type of belief—control beliefs—that could directly or indirectly combine with the two related beliefs (behavioural and normative beliefs) to predict behaviour. In particular, environmental factors or barriers might directly or indirectly predict behaviour. The factors an individual has no control over could impact intentions towards a particular behaviour. Importantly, Ajzen's conception of control beliefs parallels Bandura's self-efficacy theory (e.g., Ahmmed et al., 2014). Both control beliefs and the concept of self-efficacy provide insight into events or factors in society that might impact an individual for performing a given behaviour (Kraft et al., 2005). In this study, control beliefs and self-efficacy are used interchangeably. Here, a control belief refers to one's confidence in his or her ability to perform a given function. Overall, Ajzen (2011) argued that the three related beliefs combine to predict human behaviour by mediating through intention.

Furthermore, behaviours occur in a given context. This has contributed to the need to assess the influence of background variables on intention. According to Ajzen (1991), behaviours differ across contexts. Intention might differ between individuals and the environment. Ajzen (1991) recommended that researchers become interested in background variables (such as gender) that might influence intention towards a given behaviour. While TPB has been found to predict intentions, some authors have criticised its usefulness or sufficiency. In a conceptual paper, Morgan and Bachrach (2011) and Sommer (2011) critiqued the inability of TPB to measure past events. They argued that one's decision to give birth could be influenced by past experiences such as raising children and previous pregnancy experiences. Unfortunately, TPB measures only present events without considering what has transpired in the past. It is argued further that Ajzen focused only on the present and likely outcomes of future behaviours without considering the influence of past behaviours on intentions. There is a likelihood that what people do in the past could be repeated or have repercussions on future behaviour. Thus, it has been suggested that researchers should consider past behaviours and their influence on the future. However, in studying behaviour, what currently exists could influence future actions. This underscores the usefulness of TPB as a valid framework for studying the interaction between students with ASD and typically developing peers.

Ajzen's TPB has been operationalised to study intention towards the implementation of inclusive education (Ahmmed et al., 2014; Yan and Sin, 2014; Sharma et al., 2018; Opoku et al., 2020). For example, the aggregation of behavioural beliefs results in an attitude towards behaviour (Wang et al., 2021). First, in this study, an attitude towards behaviour was operationalised as the perspectives of typically developing students towards learning alongside peers with disabilities. Second, a normative belief accumulates into subjective norms, operationalised here as the perceived support from school leaders of all students in enhancing their learning. Third, control beliefs grow into perceived behavioural control (described in this study as self-efficacy), defined as students' confidence in their ability to perform a given task. These variables have either been studied separately or combined to assess the intention of typically developing peers towards students with ASD.

Few previous studies have used TPB to study the relationship between students with ASD and typically developing peers. For example, in the United Kingdom, Freitag and Dunsmuir (2015) examined the intentions and behaviours of typically developing primary school students towards peers with ASD. While attitudes were combined with other variables to predict intention and actual behaviour individually, the results showed no associations among attitudes, intentions, and actual behaviour. The results appear strange because Ajzen hypothesised that positive associations among the three predictors suggest that a behaviour will likely take place. There might be underlining factors in the UK explaining this result. In an earlier Australian study, Roberts and Lindsell (1997) studied the attitudes of students towards working with peers with physical disabilities. They reported the ability of attitudes and subjective norms to predict intention; however, peer attitude was found to be the strongest predictor of intention. While Roberts and Lindsell (1997) reported that subjective norms were a significant predictor of intention, its conception was unaligned with TPB. Students completed the attitude scale, but parents, teachers and principals completed the subjective norm scale. The completion of the questionnaire by different stakeholders denies us the opportunity to ascertain the influence of social pressure on students' intention. Notably, studies on the relationship between students with ASD and typically developing peers are limited in developing countries, such as Ghana. Thus, it is useful to add to the body of knowledge regarding the intentions and interactions between typically developing peers and students with ASD in Ghana. This study was guided by the following questions:

1. What is the association between demographic variables and intention (attitude, subjective norm and perceived behavioural control [PBC]) of typically developing secondary school students towards peers with ASD in Ghana?
2. Will attitudes, subjective norms and PBC combine to predict the intention of typically developing secondary school students towards learning with peers with ASD in Ghana?

## Methods

### Participants

The participants in this study were secondary school students learning alongside students with disabilities. Purposive sampling was used to select the schools, as it allowed the researchers to select participants with characteristics that were imperative for the study (Etikan and Bala, 2017). The participants were secondary school students enrolled in two inclusive schools and one non-inclusive school. The Special Education Division (SPED) of the Ghana Education Service designates inclusive schools to admit students with disabilities. In such schools, provisions for support and special education services are available for students with disabilities, including those with ASD, allowing them to access and participate in regular schools. Conversely, non-inclusive schools have yet to be formally recognised as practising inclusive education. However, they are expected to admit students with disabilities. The inclusive schools were a junior high school (JHS) (School A, 27%) and a senior high school (SHS) (School B, 55%), and the non-inclusive school was an SHS (School C, 18%). The SPED recommended schools for data collection, and they are all located in one of the 16 regions of Ghana.

Out of 900 questionnaires distributed to students, 535 were returned, representing a return rate of 59%. However, during data entry, and analysis, 10 questionnaires were found to be partially completed and were thus excluded from the analysis. During the analysis, the data were checked for outliers using boxplots since extreme values could influence results. At this stage, nine entries were deleted, leaving 516 entries for analysis. While 52% of the participants were male, 48% were female. See Table 1 for a detailed description of the participants' demographic variables.

**Table 1:** Association between demographic variables and intention.

**Category**	**Sample**	**N = 516 (%)**	**Attitudes**	**Subjective norms**	**Self-efficacy**	**Intention**
School type	School A	140 (27%)	3.27 (0.72)	2.85 (0.54)a	3.63 (0.61)a	3.50 (0.69)
	School B	284 (55%)	3.23 (0.92)	2.78 (0.52)a	3.45 (0.75)b	3.72 (1.23)
	School C	92 (18%)	3.22 (0.90)	2.53 (0.49)b	3.62 (0.57)a	3.49 (1.46)
	*F*		0.14	9.36^**^	4.09^*^	2.43
	*Partial eta*		0.001	0.04	0.02	0.01
Gender (*n* = 513)	Male	265 (52%)	3.25 (0.70)	2.76 (0.50)	3.48 (0.72)	3.50 (0.89)
	Female	248 (48%)	3.22 (0.97)	2.77 (0.54)	3.56 (0.71)	3.74 (1.8)
	*t*		0.29	−0.19	−1.32	−2.20^*^
	*partial eta*		0.001	0.001	0.003	0.01
Age (*n* = 513)	13–15 years	241 (47%)	3.18 (0.78)	2.73 (0.48)	3.49 (0.71)	3.56 (1.25)
	16–18 years	272 (53%)	3.30 (0.93)	2.79 (0.57)	3.57 (0.67)	3.68 (0.98)
	*t*		−1.50	−1.15	−1.31	−1.16
	*partial eta*		0.004	0.003	0.003	0.003
Grade Level (*n* = 511)	JHS	132 (26%)	3.28 (0.71)	2.85 (0.54)	3.61 (0.61)	3.51 (0.70)
	SHS	379 (74%)	3.22 (0.92)	2.73 (0.52)	3.48 (0.72)	3.67 (1.30)
	*t*		0.52	2.19^*^	2.02^*^	−1.69
	*partial eta*		0.001	0.01	0.009	0.006
Friend with ASD (*n* = 510)	Yes	247 (48%)	3.34 (0.90)	2.74 (0.54)	3.53 (0.67)	3.70 (1.25)
	No	263 (52%)	3.12 (0.81)	2.79 (0.51)	3.51 (0.71)	3.55 (1.10)
	*t*		2.80^**^	−0.88	0.27	1.40
	*partial eta*		0.01	0.001	0.001	0.005
Relative with ASD (*n* = 501)	Yes	151 (30%)	3.19 (0.81)	2.75 (0.60)	3.47 (0.63)	3.65 (1.28)
	No	350 (70%)	3.26 (0.88)	2.76 (0.48)	3.55 (0.71)	3.61 (1.11)
	*t*		−0.78	−0.26	−1.30	0.35
	*partial eta*		0.001	0.001	0.003	0.001

* *p < 0.05;*

** *p < 0.01;*

*ASD, Autism Spectrum Disorder; F, Analysis of variance (ANOVA); t, T-test; JHS, Junior High school; SHS, Senior High School*.

a,b *superscript means significant difference between participants*.

### Data Collection Tools

We used a survey and a four-part questionnaire for data collection from students. The survey was informed by a review of the literature that collected information about the profile of the participants. The information collected was school type, gender, age, and whether the respondent was friends with a student with ASD or had a relative with ASD.

The remaining questionnaires were selected based on components of TPB (attitudes, subjective norms, self-efficacy, and intention). The first part of the questionnaire was the Behavioural Intention Scale (BIS), developed by Selman (1980). This scale has been used in previous studies to measure intention and has reported acceptable psychometric properties (Roberts and Smith, 1999; Laws and Kelly, 2005). The scale comprises 10 items with five anchors: “1 = No; 2 = Probably no; 3 = Not sure; 4 = Probably yes; and 5 = Yes.” A mean score of exceeding four was interpreted as a high intention towards interacting with students with ASD. The items were positively worded and included “I would sit beside him/her in class,” “I would play with him/her during break time,” and “I would go to his/her house to play.”

The second part of the questionnaire measured attitudes towards peers with disabilities using a revised version of the Chedoke-McMaster Attitudes towards Children with Handicap Scale (CATCH; Rosenbaum et al., 1986). The CATCH was selected for this study because it is closely aligned with Ajzen's (1991) conception of attitudes and is one of the most commonly used scales for measuring peer attitudes towards students with disabilities (Petry, 2018). The original scale comprises 36 items. However, Bossaert and Petry (2013) shortened it to seven items for a Dutch study that was adapted for this study. The participants ranked each item on a scale ranging from 1 (strongly disagree) to 5 (strongly agree). A mean score exceeding four was deemed a positive attitude of typically developing students towards peers with ASD. Some items on the revised scale are “I would like to have a child with ASD live next door to me,” “I would be happy to have a child with ASD as a special friend,” and “I would invite a child with a disability to sleep over at my house.”

The third part of the questionnaire was the Perception of Resources Questionnaire (PRQ) (Goldan and Schwab, 2020), which was used to measure subjective norms. Of note, there is no standard scale for measuring perceived subjective norms influencing the intention of typically developing peers towards students with disabilities. Goldan and Schwab's (2020) PRQ scale measures students' perceptions of what has been provided in schools to promote inclusive practises. This scale was chosen as an ideal measure of subjective norms because we were interested in how typically developing students perceive the creation of school environments that promote the learning of students with ASD. The scale comprises 11 items and is anchored on a 4-point Likert scale, with scores ranging from 1 (not at all true) to 4 (certainly true). Goldan and Schwab (2020) described a mean score exceeding three as a higher perception of schools supporting the learning of all students. Some items on the scale are “There are enough teachers at our school,” “If we need new materials for learning, they are purchased by the school” and “Our school offers various possibilities to spend the breaks.”

The fourth part of the questionnaire was a revised General Self-Efficacy (GSE) scale adapted from Schwarzer and Jerusalem (1995). Just like subjective norms, no scale has been developed to measure control beliefs. Previous studies on intention towards inclusive practises have used self-efficacy scales to measure control beliefs (e.g., Ahmmed et al., 2014; Sharma et al., 2018). It is believed that Ajzen drew inspiration from Bandura's self-efficacy theory in his conception of control beliefs (Ahmmed et al., 2014). A self-efficacy scale was used to measure control beliefs in this study. Although the GSE scale was developed to measure an individual's general confidence, we added phrases to measure the perceived efficacy of typically developing peers towards students with ASD. This scale comprises 10 items and is ranked on a 5-point scale, with scores ranging from 1 (not at all true) to 5 (exactly true). A mean score of four in this study was interpreted as students having high confidence. The following are some items on the scale: “I can always manage to solve difficult problems I have with a student with ASD if I try hard enough,” “It is easy for me to stick to my aims to help students with ASD and accomplish my goals” and “If I am in trouble or a problem with a student with ASD, I can usually think of a solution.”

The results showed the appropriateness of the tools, as there were adequate Cronbach's alpha scores for the scales (GSE = 0.70; CATCH = 0.62; BIS = 0.70; PRQ = 0.69).

### Procedure

The study and its protocols received institutional approval before proceeding to the field for data collection. After institutional approval was received, a formal letter was sent to the SPED, which is the body supervising the implementation of inclusive education and special education. The SPED subsequently addressed a letter to the regional director of education and principals of the designated schools indicating the usefulness of the study and encouraging them to help where necessary. At the schools, the first and second authors met the principals and heads of departments and informed them about the study and the ways in which they could assist in the data collection process. In the schools, most students were below the age of consent, so letters were sent to their parents asking for consent for their children's participation. While some parents sent written notes to the research team, others opted to give oral consent. Students whose parents did not consent to participate were excluded from the study. The inclusion criteria were as follows: a student must be enrolled in any of the three schools and must have approval from parents to participate in the study if below 16 years old, the legal age of consent in Ghana.

The questionnaires were given to students who met the inclusion criteria in classrooms. They were informed about the study objective, terms were described (disability, special education, and inclusive education) and the importance of their contributions to the study was explained. Students had 2 weeks to complete the questionnaire and submit it to the research team. Data were collected from January 2018 to June 2018. Students spent ~30 min completing the questionnaires. Participation was voluntary, as the students were not reimbursed or incentivise regarding participation. They were assured that, besides the research team, their names, and school identities would remain anonymous.

### Data Analysis

The completed data from the survey and questionnaires were entered into the Statistical Package for Social Science (SPSS) version 26 before being transferred to AMOS for further analysis. Each questionnaire was checked to confirm that it had been correctly completed before entry into the software. Missing data for the scales were checked using Little's Missing Completely at Random test (Pallant, 2016). The results showed that <5% of the entries were missing at random. The expectation maximisation algorithm was used to impute the missing data. We presumed that the data were normally distributed based on Field's (2013) explanation that data from large populations are, by default, normally distributed. The internal consistencies of the scale were calculated using Cronbach's alpha. The mean scores for each scale were calculated before the study questions were answered.

To answer research question 1, a *t*-test, and analysis of variance were calculated to ascertain the associations between demographic variables and predictors of intention. While a *t*-test was calculated for demographics with two levels (e.g., age), ANOVA was computed for demographics with three levels (e.g., school type). When there was a significant difference between demographics and predictors, a *post-hoc* comparison using Tukey's HSD test was computed to cheque where the differences may lie (Pallant, 2016). The assumption of homogeneity of variance was checked using Levene's test to ensure there was no violation (Pallant, 2016).

**Figure 1:**
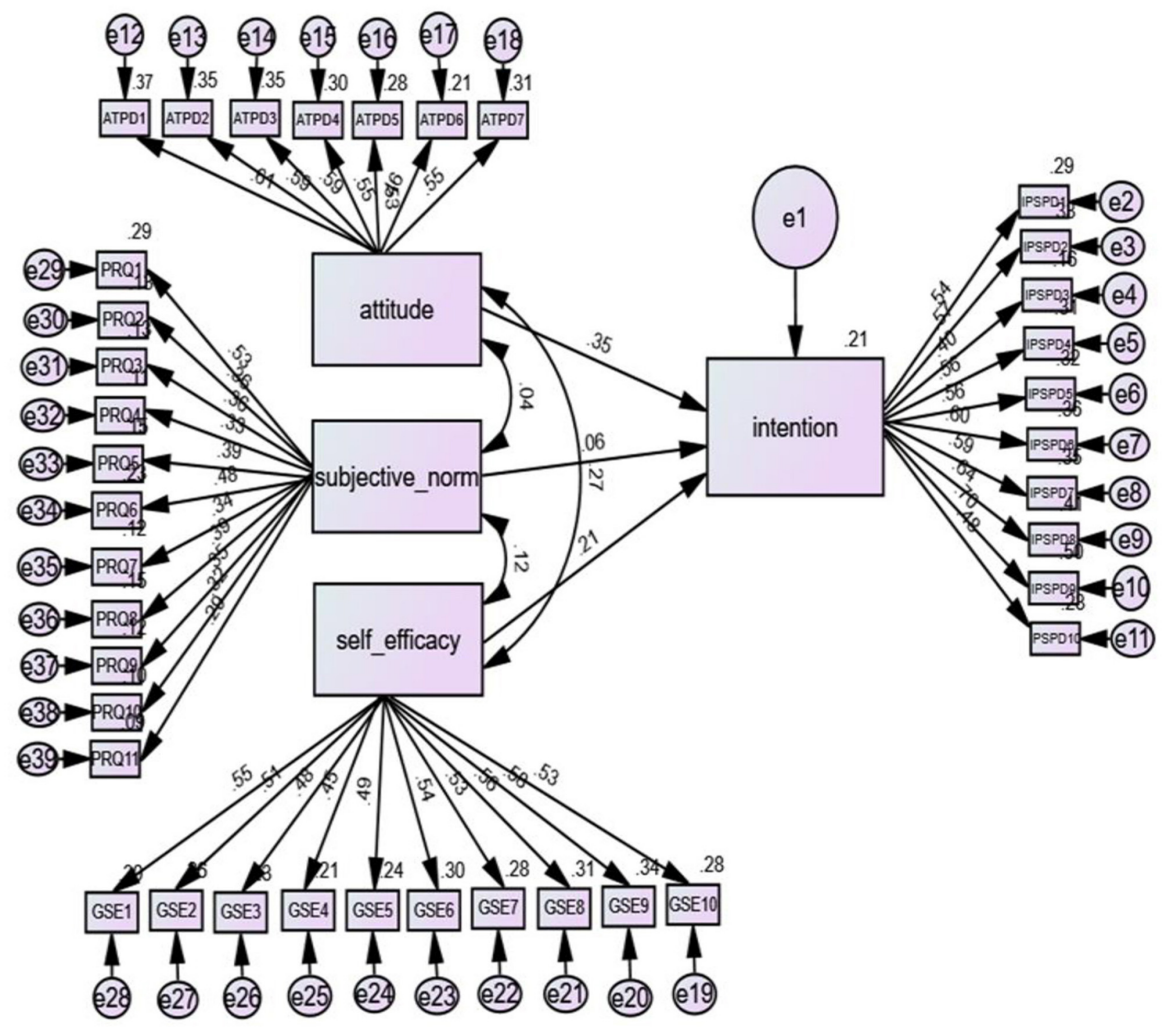
Predictive Pathways From SEM.

To answer research question 2, we used structural equation modelling (SEM) to ascertain the predictive utility of TPB using AMOS (Figure 1). Specifically, confirmatory factor analysis was performed to ascertain whether attitude, subjective norms and self-efficacy would combine to predict intentions. The data were transferred from SPSS to AMOS for this computation. The maximum likelihood method was used to assess the appropriateness of the estimated algorithm. We checked the fitness of the SEM for this study using chi-square (χ^2^) and the goodness-of-fit index (GFI), comparative fit index (CFI) and the standardised root-mean-square error of approximation (RMSEA). The correlations between the predictors were also observed and interpreted as follows: small, *r* = 0.10 to 0.29; medium, *r* = 0.30 to 0.49; and large, *r* = 0.50 to 1 (Pallant, 2016).

## Results

The mean scores showed that students were ambivalent on all the scores: attitudes towards students with disabilities (*M* = 3.24; *SD* = 0.86), subjective norms (*M* = 2.76; *SD* = 0.53), self-efficacy (*M* = 3.53; *SD* = 0.69) and intention towards learning alongside students with disabilities (*M* = 2.76; *SD* = 0.53).

### Impact of Demographics on Attitude, Subjective Norms, Self-Efficacy, and Intention

The associations between demographics and attitudes, subjective norms, self-efficacy, and intention were assessed using *t*-tests and ANOVAs (see Table 1 for details). Regarding the *t*-test, there was a significant relationship between participant: gender, grade level, and having a friend with a disability. In gender, there was a significant difference between male and female students on intention only, with the latter being more positive than the former [*t*_(489)_ = −2.23, *p* = 0.03]. However, the effect size was miniscule, *partial eta squared* (*n* = *0.01*).

Regarding grade level, there was a significant relationship between groups in subjective norms and self-efficacy, with small effect sizes. Regarding subjective norms, students who indicated they were in JHS indicated a higher perception of the availability of support and services to enhance students' learning than their counterparts in SHSs [*t*_(458)_ = 2.19, *p* = 0.03], *partial eta squared* (*n* = *0.01*). Similar trends were observed regarding self-efficacy [*t*_(489)_ = 2.02, *p* = 0.05], *partial eta squared* (*n* = *0.009*), with JHS students more confident about supporting students with ASD than SHS students. Regarding having a friend with ASD, students who indicated they had such friends had more positive attitudes than those who indicated otherwise [*t*_(479)_ = 2.80, *p* = 0.005], *partial eta squared* (n = 0.01).

For the ANOVA computations, there was a significant relationship between the participants regarding school types in subjective norms, and self-efficacy. Students who were in an inclusive JHS (School A) indicated that they received more support than those who were in an inclusive SHS (School B) or non-inclusive SHS (School C) [*F*_(2, 474)_ = 9.36, *p* = 0.001], partial eta squared (n = 0.04). *Post-hoc* comparisons showed a significant difference between School A and School C. However, there was no difference between the participants in School A and School B. For self-efficacy, students in School A were more confident than students in School B and School C [*F*_(2, 506)_ = 4.09, *p* = 0.02], partial eta squared (*n* = 0.02). *Post-hoc* comparisons using Tukey's HSD test showed significant differences between participants from School A and School B (*p* < 0.03). Conversely, there was no significant difference between School A and School C.

### Predictors of Intention

We evaluated the predictors of TPB using AMOS (Figure 1). The first run results showed an acceptable range, which could support the conclusion that the observed data were appropriate. The evaluation of the results showed that the data reached acceptable fit indices: χ^2^ [148] = 108.06; χ^2^/df = 1.91; CFI = 1; GFI = 0.96; RMSEA = 0.16. Significant pathways were observed between the predictors and intention towards students with ASD: attitude and intention (*beta* = 0.35, *p* = 0.001), subjective norms and intention (*beta* = 0.06, *p* = 0.05) and self-efficacy and intention (*beta* = 0.21, *p* = 0.001). The predictors contributed 21% of the variance in intention. While subjective norm and self-efficacy positively predicted intention, attitude made the largest contribution to the variance in intention.

The convergent validity of the constructs was examined by observing the correlations between the predictors. There was a small but positive correlation between attitude and self-efficacy (*r* = 0.27, *p* = 0.001) and between subjective norms and self-efficacy (*r* = 0.12, *p* = 0.05). However, there was no significant relationship between attitude and subjective norms (*r* = 0.04, *p* = 0.45).

## Discussion

The complexity of the factors influencing the implementation of inclusive education cannot be overemphasised. This seems to have contributed to recent interest in using TPB (Ajzen, 1991) as a theoretical framework to assess intention towards the implementation of inclusive education. Ajzen conceptualised intention towards a behaviour as a product of three interrelated beliefs. Indeed, the results of this study confirm Ajzen's (1991) conceptualisation of TPB as attitude, subjective norms, and PBC combined to predict intention. This finding parallels that of previous studies that reported that the three related beliefs combine to predict intention towards peers with disabilities (Roberts and Smith, 1999; Freitag and Dunsmuir, 2015). This seems to suggest that changing attitudes, providing resources and developing students' confidence are vital to promoting the acceptance of students with ASD among typically developing peers. This is critical in the Ghanaian context because of the misconceptions and stereotypes of individuals with ASD, who are seen as “spirits” and victims of mockery in society (Ametepee and Anastasiou, 2015).

In previous study, Wang et al. (2021) noted that positive attitude of students enable them to develop effective interpersonal relationship in the classroom. Attitude towards peers with ASD emerged as a significant predictor of intention. This finding parallels that of previous studies that revealed attitude as a significant contributor to the variance in intention of typically developing students towards peers with disabilities (Roberts and Lindsell, 1997). This is unsurprising because developing a positive attitude towards inclusion seems to be a first step towards practising inclusion (De Boer et al., 2011). Therefore, attitudes towards students with ASD might need to be changed in a hostile environment. In general, individuals with disabilities, such as ASD, are not supported in Ghanaian society with access to essential services (Kassah et al., 2014; Opoku et al., 2019). They are likely to be rejected, side-lined, and discriminated against for accessing essential services, such as education. Such treatment mainly results from cultural beliefs that attribute disability to supernatural and superstitious sources, such as curse by deity, punishment for one's wrongs and possession by evil spirits (Anthony, 2011). It is apparent that changing attitudes regarding the acceptance of children with ASD is a primary step towards enhancing the equal participation of all in activities in society. In this way, they would be embraced as equal members of the school and supported in learning or developing independent living skills. Developing good relationships between typically developing students and peers with ASD can enhance the retention of the latter in regular classrooms. ASD students can then benefit academically and socially from their inclusion in regular schools.

Positive correlations among attitude, subjective norms and PBC affect intention towards behaviour (Ajzen, 1991). However, this study did not support Ajzen's hypothesis, as there was no significant relationship between attitude and subjective norm. This finding disagrees with that of previous studies that have reported positive correlations between all predictors in inclusive research (Roberts and Smith, 1999; Ahmmed et al., 2014; Yan and Sin, 2014). The results suggest that students' attitudes towards peers with ASD may not depend on the support initiatives or steps taken by school leaders to promote the acceptance of students with ASD in regular classrooms. This is possible because, in the Ghanaian context, there is a lack of resources and poor interventions in schools to promote inclusive practises (Opoku et al., 2021). Clearly, school leaders and teachers could probably not create an environment conducive to the participation of all students (Mantey, 2017). School leaders and teachers cannot lead advocacy for the implementation of inclusive education because of the government's inability to provide schools with the funding and other resources needed to practise inclusivity (Subbey, 2020). Perhaps there is an absence of programmes aimed at creating awareness among students regarding acceptance and assisting with the learning needs of peers with ASD. Although typically developing peers might accept peers with ASD as equals in schools without support from school leaders and teachers, such assistance could be consolidated if the school system provides for the participation of all students.

The results show some background variables that seem to provide additional explanations about the intention of peers towards ASD. Based on Ajzen's (1991) suggestion, we assessed the influence of background variables on attitudes, subjective norms and PBC. For instance, female students were found to be more positive in intention than male students. This finding probably suggests that female students will be more open to supporting students with ASD than their male counterparts; this finding partly agrees with that of previous studies that have reported the likelihood of female students interacting with students with disabilities (Laws and Kelly, 2005). This finding is also unsurprising in the Ghanaian context because of the existence of traditional gender roles. For example, women are assigned traditional caregiving and domestic duties, while men are expected to provide food and shelter (Ferrant et al., 2014). Female students have perhaps been raised to perform caregiving and motherly roles and thus may have a cultural inclination towards showing empathy towards vulnerable groups, such as individuals with ASD. However, in contemporary society, there are discussions about neutralising gender roles in Ghana, especially the way rigid gender roles appear to overburden women with responsibility, causing stress and the inability to occupy influential positions in society. Caregiving should be the responsibility of all, and, as such, both male, and female students could be trained to contribute to the development of all.

Another background variable that provides additional insight into attitude is having a friend with ASD. Positive relationships between typically developing students and peers with ASD positively affect attitudes. Specifically, having a friend with ASD was found to influence attitudes towards peers with ASD. Participants who reported having friends with ASD had more positive attitudes than those who indicated otherwise. This finding partly corroborates the results of previous studies reporting that students who had friends with ASD were more receptive to and favourable towards them than those who did not (Mavropoulou and Sideridis, 2014). This is not unexpected because participants who have friends with ASD might understand their uniqueness and needs. Developing positive relationships between students with ASD and typically developing peers is a step in the right direction in Ghana. This can affect the self-worth, confidence and participation of persons with ASD in productive activities, such as education. Inclusive education aims to foster cohesion, acceptance, and opportunities for all persons in society (UNESCO, 1994). As part of an effort to promote inclusion in Ghana and to tolerate and celebrate diversity, policymakers, and educators could encourage interactions among students to achieve the intended purpose. This could be a strategy that policymakers and teachers in Ghana could adopt to promote acceptance between typically developing students and peers with ASD.

### Study Limitations

The results of this study should be interpreted with caution because of many limitations. First, the schools chosen for this study were nominated by the SPED; thus, they are likely influenced by bias. All the schools were also located in one region, which might limit their ability to generalise from the study findings. Schools in Ghana use the same curricula, facilities and teachers with similar qualifications or conditions of service. Therefore, it is possible that the situation in one region could be comparable to that in other regions. However, it is important for future studies to compare the intentions of typically developing peers across regions and to compare them with the findings reported here. Nevertheless, the students were educated about the study objectives, and those who participated in this study had ideas about disability, segregation, and inclusive education. Also, like in all quantitative studies, voices providing detailed insight into the results were undocumented. While this study has provided some information about the interactions between students with ASD and typically developing peers, it was beyond the scope of this study to interview students and document their perspectives. Future studies could adopt qualitative methods for developing deeper insights into the intention of students. This study also assessed the intention of typically developing peers towards students with ASD only. One component of the theory, the influence of predictors, and intention on actual behaviour, was unassessed. We recommend that future studies adopt experimental designs to observe actual interactions between typically developing students and peers with ASD to ascertain whether intention directly influences actual behaviour. The major strengths of this study were the collection of data from different schools and the large sample size and return rate. Therefore, these findings could be considered a step towards promoting equitable access to education for students with ASD.

## Conclusion and Study Implications

This study presents the results of an exploratory study that assessed the intention of typically developing students towards learning in the same classroom alongside peers with ASD. More importantly, this seems to be the first study in a developing country to test TPB ability to predict the intention of typically developing students towards learning alongside peers with ASD. The study is highly relevant, as secondary school education has been identified as the next frontier in implementing inclusive education. The findings of this study support Ajzen's (1991) conception that synergising attitudes, subjective norms and PBC predict the intention of typically developing peers towards students with ASD. While there were correlations between intention and predictors and among predictors, there was no relationship between subjective norms and attitudes. Other demographic variables, such as gender, contact with friends with ASD, school type and grade level, influenced the intention of students towards peers with ASD. The behavioural needs of students with ASD (American Psychiatric Association, 2013) probably underscore the need for policymakers to consider the findings of this study regarding school reform to promote inclusive practise.

This study's findings have implications for policymaking in Ghana and other similar contexts that are at the early stages of implementing inclusive education. ASD is poorly understood and supported in especially sub-Saharan African context (Anthony, 2011), which provides an impetus for educators to take steps to encourage equitable participation. Specifically, this study's results perhaps justify the need for policymakers to invest in programmes geared towards changing attitudes, promoting school support and building the self-efficacy of typically developing peers towards students with ASD. For instance, the results may underscore the need for policymakers to expedite attitudinal change campaigns and develop programmes to enhance mutual respect between typically developing students and peers with ASD. In this way, typically developing students can attain positive attitudes and be aware of provisions made for the participation of students with ASD in regular classrooms. This campaign could be expanded to the community, where the masses could be educated to embrace persons with ASD as equal members of society. Also, education and social interventions have been considered useful in developing positive relationships between typically developing students and peers with ASD (Boutot, 2007; Locke et al., 2010). Policymakers and teachers could organise educational programmes and adopt strategies to encourage typically developing students to befriend their peers with ASD in regular schools. Class meetings could be organised so that students who have had friends with ASD can speak to the class about what the students with ASD are good at and their likes, interests, hobbies or sports prior to the arrival of the student with ASD to prepare typically developing peers. This would promote understanding, acceptance, friendships and even the retention of students with ASD in regular schools.

## Data Availability Statement

The original contributions presented in the study are included in the article/supplementary material, further inquiries can be directed to the corresponding author/s.

## Ethics Statement

The studies involving human participants were reviewed and approved by Kwame Nkrumah University of Science and Technology. Written informed consent to participate in this study was provided by the participants' legal guardian/next of kin.

## Author Contributions

MO and EA-O collected the data. MO and WN analysed the data. EA-O, J-F, WM, and MO reviewed literature and drafted the initial draft of the manuscript. WN, J-F, and WM made intellectual contribution to revised manuscript. All the authors read and consented to submission of the final draft and participated in the conception of the study.

## Conflict of Interest

The authors declare that the research was conducted in the absence of any commercial or financial relationships that could be construed as a potential conflict of interest.

## Publisher's Note

All claims expressed in this article are solely those of the authors and do not necessarily represent those of their affiliated organizations, or those of the publisher, the editors and the reviewers. Any product that may be evaluated in this article, or claim that may be made by its manufacturer, is not guaranteed or endorsed by the publisher.
